# Patient Reported Experiences and Delays During the Diagnostic Pathway for Pulmonary Fibrosis: A Multinational European Survey

**DOI:** 10.3389/fmed.2021.711194

**Published:** 2021-08-04

**Authors:** Iris G. van der Sar, Steve Jones, Deborah L. Clarke, Francesco Bonella, Jean Michel Fourrier, Katarzyna Lewandowska, Guadalupe Bermudo, Alexander Simidchiev, Irina R. Strambu, Marlies S. Wijsenbeek, Helen Parfrey

**Affiliations:** ^1^Erasmus Medical Center, Rotterdam, Netherlands; ^2^Action for Pulmonary Fibrosis, Lichfield, United Kingdom; ^3^Galapagos NV, Mechelen, Belgium; ^4^Ruhrlandklinik, University of Duisburg-Essen, Essen, Germany; ^5^Association Pierre Enjalran Fibrose Pulmonaire Idiopathique, Meyzieu, France; ^6^Department of Pulmonary Diseases, National Research Institute of Tuberculosis and Lung Diseases, Warsaw, Poland; ^7^Hospital Universitari de Bellvitge, Barcelona, Spain; ^8^Department of Functional Diagnostics, Medical Institute MVR, Sofia, Bulgaria; ^9^Carol Davila University of Medicine and Pharmacy, Bucharest, Romania; ^10^Royal Papworth Hospital, Cambridge, United Kingdom

**Keywords:** pulmonary fibrosis, delayed diagnosis, diagnostic journey, survey, patient reported outcomes

## Abstract

**Introduction:** Pulmonary fibrosis includes a spectrum of diseases and is incurable. There is a variation in disease course, but it is often progressive leading to increased breathlessness, impaired quality of life, and decreased life expectancy. Detection of pulmonary fibrosis is challenging, which contributes to considerable delays in diagnosis and treatment. More knowledge about the diagnostic journey from patients' perspective is needed to improve the diagnostic pathway. The aims of this study were to evaluate the time to diagnosis of pulmonary fibrosis, identify potential reasons for delays, and document patients emotions.

**Methods:** Members of European patient organisations, with a self-reported diagnosis of pulmonary fibrosis, were invited to participate in an online survey. The survey assessed the diagnostic pathway retrospectively, focusing on four stages: (1) time from initial symptoms to first appointment in primary care; (2) time to hospital referral; (3) time to first hospital appointment; (4) time to final diagnosis. It comprised open-ended and closed questions focusing on time to diagnosis, factors contributing to delays, diagnostic tests, patient emotions, and information provision.

**Results:** Two hundred and seventy three participants (214 idiopathic pulmonary fibrosis, 28 sarcoidosis, 31 other) from 13 countries responded. Forty percent of individuals took ≥1 year to receive a final diagnosis. Greatest delays were reported in stage 1, with only 50.2% making an appointment within 3 months. For stage 2, 73.3% reported a hospital referral within three primary care visits. However, 9.9% reported six or more visits. After referral, 76.9% of patients were assessed by a specialist within 3 months (stage 3) and 62.6% received a final diagnosis within 3 months of their first hospital visit (stage 4). Emotions during the journey were overall negative. A major need for more information and support during and after the diagnostic process was identified.

**Conclusion:** The time to diagnose pulmonary fibrosis varies widely across Europe. Delays occur at each stage of the diagnostic pathway. Raising awareness about pulmonary fibrosis amongst the general population and healthcare workers is essential to shorten the time to diagnosis. Furthermore, there remains a need to provide patients with sufficient information and support at all stages of their diagnostic journey.

## Introduction

Interstitial lung disease (ILD) describes a relatively uncommon group of diseases characterised by inflammation and fibrosis of the lung interstitium. Pulmonary fibrosis is a chronic, and often progressive condition. There is, however, considerable variation amongst patients in terms of aetiology, treatment strategies, and disease course (1). Amongst all types of pulmonary fibrosis, idiopathic pulmonary fibrosis (IPF) is the most prevalent and accounts for about two-thirds of cases. It has the worst prognosis due to rapid disease progression with a mean survival of 4 years from diagnosis without anti-fibrotic therapy (2). Other types of progressive pulmonary fibrosis include chronic hypersensitivity pneumonitis, auto-immune disease related ILD, and occupational diseases such as asbestosis (1). Epidemiological data for all types of pulmonary fibrosis are limited as most registries and studies have focused on IPF or progressive phenotypes only (3). The reported prevalence (per 100,000 persons) of the ILDs that most often result in pulmonary fibrosis is 30.2 for sarcoidosis, 12.1 for ILD related to a connective tissue disease and 8.2 for IPF. Overall, the proportion of ILD patients who develop pulmonary fibrosis varies from 13 to 100% per individual disease (1).

The diagnostic journey usually starts with patients presenting to their primary care physicians with initial symptoms of cough or mild dyspnoea. These non-specific symptoms, combined with the heterogeneity, and rarity of pulmonary fibrosis, as well as requirement for multiple diagnostic investigations, results in a prolonged time to diagnosis with potential delays related to patient factors and healthcare systems (4). Reported time to diagnosis from the onset of initial symptoms varies in different studies but may be up to a median of 2.1 years (IQR 0.9–5.0) (5). Longer time to diagnosis is associated with worse outcomes in IPF (6, 7), causes delayed treatment, leads to more extensive fibrosis (8) and affects patients' well-being. Therefore, it is important to get better insights into patients' experiences during the diagnostic journey to identify reasons for potential delays. Understanding patients' experiences will also help healthcare workers guide and support patients during their diagnosis journey. However, to date, only a few studies have explored the reasons for diagnostic delays using data reported by pulmonary fibrosis patients (9–13). Most analyses are based on retrospective data obtained from healthcare records (5, 7, 8, 14–18).

In this paper, we present data obtained from a multinational patient survey regarding time to diagnosis and potential causes for diagnostic delays, together with patient experiences on the pathway to diagnosis. Based upon these findings, we provide general recommendations to improve the diagnostic process.

## Methods

### Survey Design and Distribution

**Figure 1:**
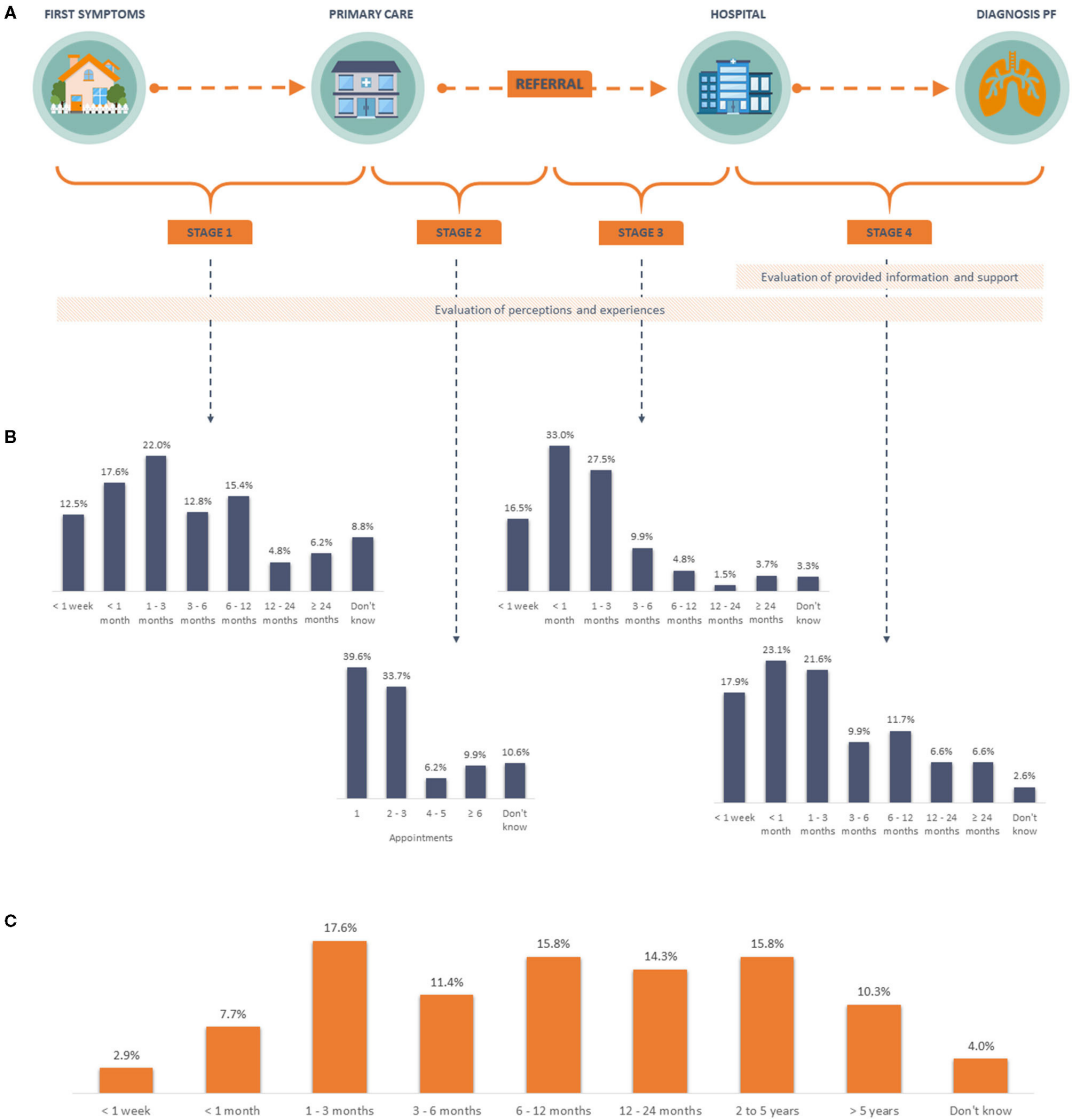
Diagnostic pathway and time to diagnosis. **(A)** Schematic overview of the diagnostic pathway for pulmonary fibrosis, including stages and topics assessed in the survey. **(B)** Patient reported time per stage. **(C)** Patient reported overall time to diagnosis. PF, pulmonary fibrosis.

A survey was designed to collect quantitative and qualitative data from patients diagnosed with pulmonary fibrosis across Europe. This survey was developed based upon a market research survey on the IPF patient journey (unpublished data) carried out using a mixture of in-depth telephone interviews with 28 patients and 30 pulmonologists, and online interviews with 315 pulmonologists spanning USA, France, Germany, Italy, Spain, United Kingdom, Australia, Brazil, Canada, and Japan. The patient survey was developed jointly between Galapagos and two patient organisations: Action for Pulmonary Fibrosis (APF, based in the United Kingdom) and the European Idiopathic Pulmonary Fibrosis and Related Disorders Federation (EU-IPFF). Insights from this patient journey research resulted in a questionnaire incorporating both closed and open-ended questions, which focused on the following four stages of the patient journey to identify key points in the delay to diagnosis. The first stage was the time from first onset of symptoms at home, before seeking medical attention in a primary care setting; the second the amount of visits in primary care before being referred to a hospital specialist; the third the time taken to be seen in a hospital by a specialist; and the last the time taken to receive a diagnosis (Figure 1A). The survey also gathered data on the overall time from first onset of symptoms to diagnosis and information provided by healthcare workers. Patients were also asked about their feelings throughout the diagnostic journey and to provide advice for patients navigating this journey in the future. No personalised data were collected and all data were anonymised. The questionnaire was designed in English and translated into seven languages (Bulgarian, Dutch, French, German, Hungarian, Italian, and Spanish) by a certified translation agency. It was created using the Typeform® platform. Patients were invited to complete the questionnaire by an e-mail containing a link to the platform. The complete survey in English can be found in the Supplementary Material 1.

The survey was disseminated by the EU-IPFF through its member patient organisations in Europe; these organisations distributed the survey to members and other patients through email and social media. Patients with a self-reported diagnosis of pulmonary fibrosis, and who had an email address and internet access were eligible to participate. The survey was sent out on 7th June 2020 with a reminder after 2 weeks. It closed on 1st July 2020. Ethical review was not required for this online questionnaire. Patients agreed with the use of their responses for further analysis without collection of personal data and were informed that all data was anonymised.

### Data Analysis

Responses in languages other than English were translated into English by a certified translation agency. Open-ended questions were assessed qualitatively and coded or categorised for interpretation. Data were uploaded and calculations were performed in Excel (Microsoft, Redmond, WA, USA). R version 4.0.3 for Mac OS X GUI (PBC, Boston, MA, USA) was used for creating a word cloud. All responses were included in the analysis, except for blank responses.

### Literature Search

In addition to the survey, a literature search on diagnostic delays in ILD, with a focus on pulmonary fibrosis, was conducted in order to provide a complete overview of the available evidence from patient surveys, physician surveys, and medical file analysis.

The systematic literature search was performed in Embase, Medline, Web of science, Cochrane, and Google scholar databases. The following search terms were used: diagnostic delay, time to diagnosis, interstitial lung disease (including sarcoidosis, vasculitis, interstitial pneumonia). Full search and outcome can be found in the Supplementary Material 2. Animal studies, paediatric subjects and articles in languages other than English were excluded. The reference list was screened for relevance by title and abstract. Letters to the editor, abstracts, posters, and articles without available full text were excluded.

## Results

### Respondent Characteristics

**Figure 2:**
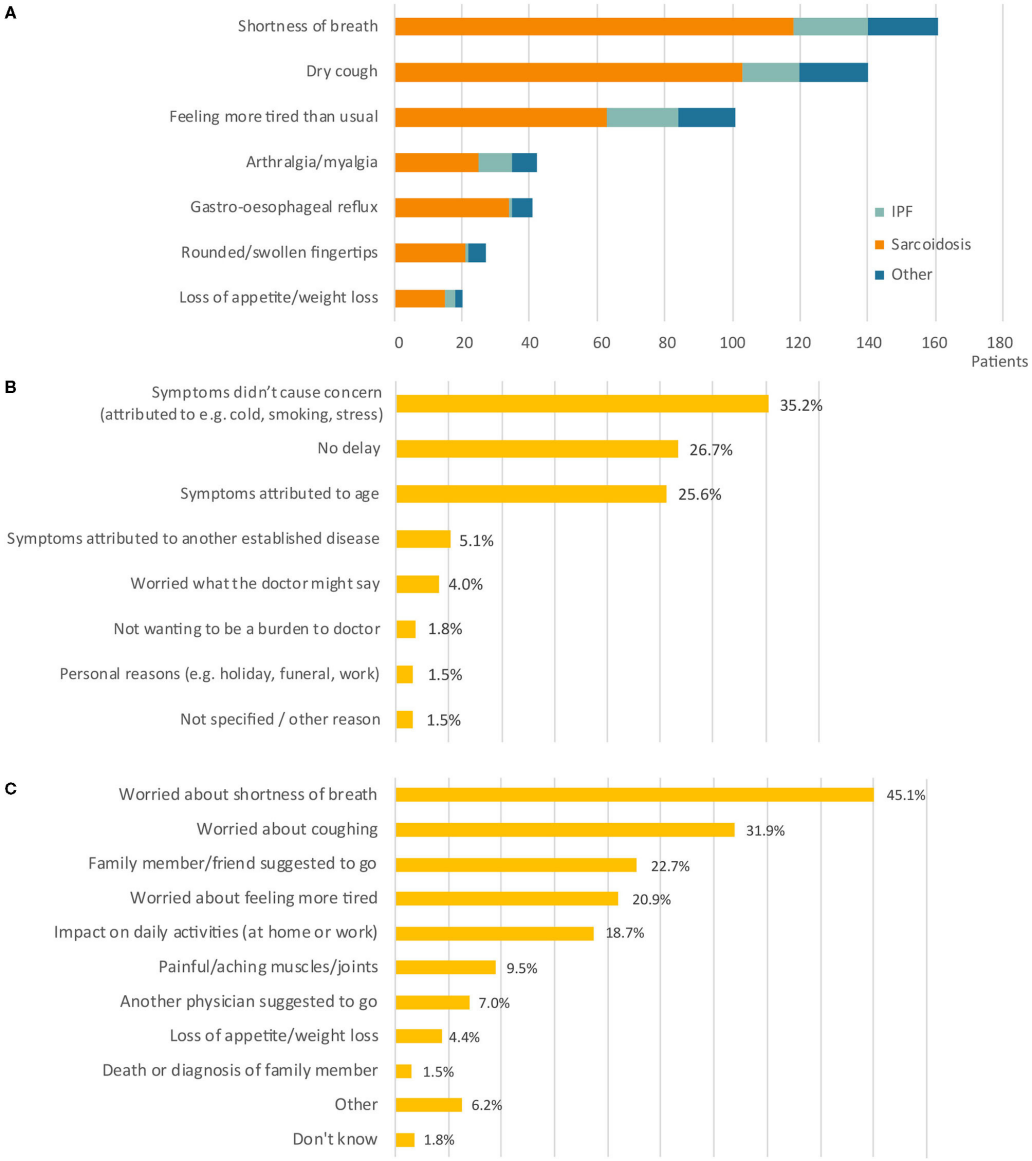
Patient symptoms and motives in stage 1. **(A)** Number of patients (*n* =) reporting a specific symptom at onset. Bars are divided into diagnosis groups (total responses *n* = 532). **(B)** Reason to delay the initial primary care appointment (*n* = 277). **(C)** Reason to schedule the initial primary care appointment (*n* = 463). Percentages do not add up to 100% as more than one response was allowed. IPF, idiopathic pulmonary fibrosis.

Two hundred and seventy three patients from thirteen different countries responded. The largest group of respondents were IPF patients (*n* = 214, 78.4%), followed by sarcoidosis (*n* = 28, 10.3%). Other types of pulmonary fibrosis diagnoses accounted for 31 respondents (11.4%) and included patients with autoimmune related disorders, chronic hypersensitivity pneumonitis, and other conditions. The majority of respondents received a diagnosis of pulmonary fibrosis in Spain (21.6%), Belgium (20.1%), United Kingdom (18.3%), Italy (17.2%), or Germany (10.6%). A smaller number of respondents were diagnosed in the Netherlands (3.3%), Bulgaria (2.6%), France (1.8%), Poland (1.8%), Austria (1.5%), Ireland (0.4%), Norway 0.4%), and Romania (0.4%). Shortness of breath, dry cough, and tiredness were the most common initial symptoms in all diagnosis groups (Figure 2A).

The total time from initial symptom onset to a final diagnosis of pulmonary fibrosis, varied greatly amongst patients (Figure 1C). Overall, nearly 30% received a diagnosis within 3 months, with 31.3% patients with IPF receiving a diagnosis within 3 months, compared to 14.3% for sarcoidosis and 19.4% for other types of pulmonary fibrosis. Moreover, 40.2% of all patients had to wait a year or more to be diagnosed, with the largest difference between the proportion of patient with IPF (36.4%) and other types of pulmonary fibrosis (58.1%).

### Stages of the Diagnostic Process

#### Stage 1: From Initial Symptom Onset to First Primary Care Assessment

More than half of respondents made a first appointment with a primary care physician within 3 months of symptom onset (52.0%), but nearly 30% waited more than 6 months (Figure 1B, stage 1). A number of patients responded that they did not delay visiting their doctor (26.7%).

Of all patients with a delay in stage 1 of 6 months or less (*n* = 177), 65.0% reported a total time to diagnosis of 1 year or less. Where patients with a delay of more than 6 months (*n* = 72) in this stage, only 34.7% reported being diagnosed within a year.

There were a variety of reasons for delays (Figure 2B). In a large number of cases, patients delayed seeking medical advice because they were not concerned about their symptoms. Patients believed symptoms were related to other causes (e.g., cold, smoking, stress; 35.2%), related to age (25.6%), or due to another established disease (5.1%). The main reasons that triggered patients to make an appointment with their primary care physician were worries about their symptoms, including shortness of breath (45.1%), cough (31.9%), and fatigue (20.9%) (Figure 2C). For 18.7% of patients, it was the impact of symptoms on their daily activities, especially on physical activity (e.g., sports, climbing stairs, walking, household, gardening) and work-related activities that led them to consult their primary care physician. In addition, some patients were prompted to make an appointment following the suggestion from family members or friends (22.7%), or another physician (7%).

#### Stage 2: From Start of Primary Care Assessment to Referral to Pulmonologist

**Figure 3:**
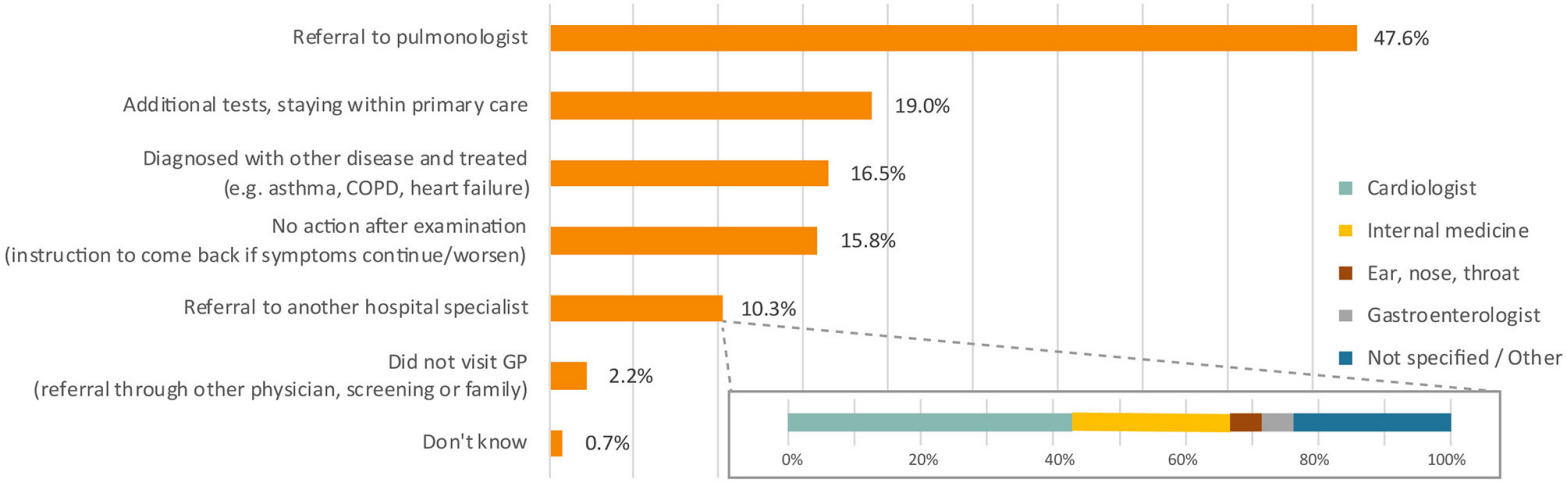
Action of physician at first visit primary care. Percentages do not add up to 100% as more than one response was allowed. Total responses *n* = 306. COPD, chronic obstructive pulmonary disease; GP, general practitioner.

At the first primary care appointment, a variety of actions were taken by the treating physicians. Almost half of all patients were referred to a pulmonologist (Figure 3). Other reported physician's actions included additional tests (19.0%), treatment for another disease (16.5%), and referral to other specialists rather than a pulmonologist (10.3%). Overall, the majority (73.3%) of patients were referred to a pulmonologist within three primary care visits, but for 9.9% of patients it took six or more appointments (Figure 1B, stage 2).

Comparing the different diagnosis groups, 43.2% of IPF patients were referred to a pulmonologist after one primary care visit. This was lower for those with sarcoidosis (28.6%) and other types of pulmonary fibrosis (25.8%). Furthermore, 39.3% of sarcoidosis patients were referred after six or more primary care visits, compared to 6.6% of IPF and 6.7% of other fibrosis types in this cohort.

#### Stage 3: From Referral to First Hospital Appointment

Once patients were referred to a pulmonary specialist, 76.9% of all patients had their first visit within 3 months (Figure 1B, stage 3). This was lower for the subgroup of sarcoidosis patients (50.0%) compared to IPF (79.9%), and other types of pulmonary fibrosis (80.6%). Few IPF patients (2.3%) had a delay of more than a year from referral to first hospital appointment, in contrast to almost a third of the sarcoidosis patients (32.1%). All patients with other types of pulmonary fibrosis were assessed within a year of the referral.

#### Stage 4: From First Hospital Appointment to Diagnosis Pulmonary Fibrosis

The 273 respondents underwent a total of 1,232 diagnostic tests in the hospital (Table 1). The majority of patients reported having performed spirometry (*n* = 246), blood tests (*n* = 222) and chest imaging (X-ray *n* = 209; CT scan *n* = 201) without large differences in proportions between the diagnosis subgroups. Other tests reported included assessment of 6-min walk test (*n* = 149), lung biopsy (*n* = 125), and bronchoaveolar lavage (*n* = 74). Lung biopsy was more frequently reported by sarcoidosis patients compared to the other subgroups.

**Table 1:** Performed tests in hospital before diagnosis.

	**IPF (** ***n*** **=** **214)**		**Sarcoidosis (** ***n*** **=** **28)**		**Other type (** ***n*** **=** **31)**	
**Tests**	***n* =**	**% of patients in subgroup**	***n* =**	**% of patients in subgroup**	***n* =**	**% of patients in subgroup**
Spirometry	194	90.7%	24	85.7%	28	90.3%
Blood tests	168	78.5%	26	92.9%	28	90.3%
Chest X-ray	161	75.2%	22	78.6%	26	83.9%
CT scan	156	72.9%	19	67.9%	26	83.9%
6-min walk test	120	56.1%	10	35.7%	19	61.3%
Lung biopsy	93	43.5%	19	67.9%	13	41.9%
Bronchoaveolar lavage	49	22.9%	11	39.3%	14	45.2%
Other/Don't know	5	2.3%	1	3.6%	-	-
*Tests per patient (mean)*	*4.4*		*4.7*		*5.0*	

*Number of patients (n =) reporting a specific diagnostic test. Percentages do not add up to 100% as more than one response was allowed. CT, Computed tomography; IPF, idiopathic pulmonary fibrosis*.

Although the final diagnosis was made within 3 months of the first hospital appointment for 62.6% of the 273 patients (Figure 1B, stage 4), 21.6% took between 3 months and 1 year, and 13.2% took over 1 year; 2.6% did not know how long this took. Small differences were found between the proportion of patients in each diagnosis group who were diagnosed within 3 months (IPF 64.5%, sarcoidosis 50.0%, and other pulmonary fibrosis types 61.3%) and more than 1 year after the first hospital appointment (IPF 11.2%, sarcoidosis 21.4%, and other pulmonary fibrosis types 19.4%).

### Experiences and Recommendations

#### Information Provision

We assessed the patient perceptions on the information provided at the different stages in the diagnostic pathway. During assessment at the hospital (stage 4), 13.6% of patients reported not knowing why certain diagnostic tests were being performed. Almost a quarter (23.6%) of all patients felt they received insufficient information. At diagnosis, most patients (75.6%) received an explanation about their diagnosis from a physician and/or specialist nurse during a consultation. However, only 6.0% percent of patients received educational materials and 6.0% received information related to support groups. A small number (3.0%) reported not having received any information at the time of diagnosis. In response to an open-ended question, patients reported that the discussion with their doctor or nurse was particularly valuable, as well as ongoing follow up appointments at the hospital and contact details to enable them to ask questions or reach out if they were feeling unwell.

The patients stated that they would have benefitted from more information during the diagnostic process, not only after the diagnosis was established. They would have welcomed more information before, at and after diagnosis on the following topics: differential diagnosis, diagnostic tests, available pharmacological, and non-pharmacological therapies, disease course, and prognosis. Respondents would have also liked more information on living with pulmonary fibrosis day-to-day, future perspectives, access to a psychologist, and information on peer support groups for patients and carers.

#### Emotional Experiences

**Figure 4:**
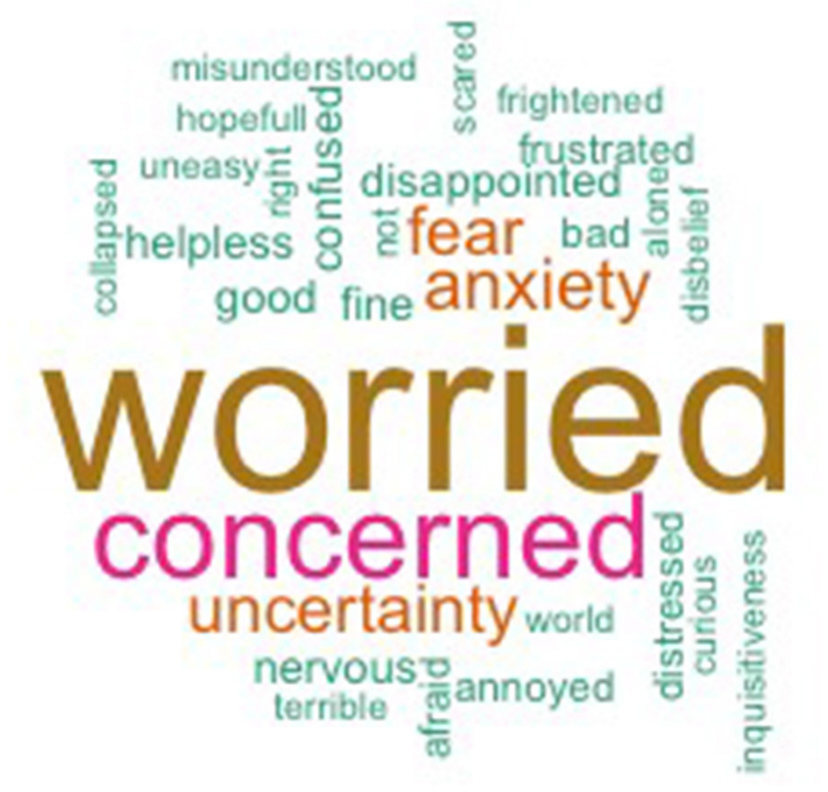
Reported feelings during stage 3. Words grouped after coding, ones with minimum frequency of 2 are included in figure (*n* = 28). Full list (*n* = 62) can be found in Supplementary Material 3.

Patients' perceptions and experiences were retrospectively assessed at different time points during their diagnostic journey. When describing their feelings after the onset of symptoms before their first doctor's visit (*n* = 179 responses), 65.4% of the respondents experienced negative emotions, 5.6% positive emotions, and the remainder (29.1%) were neutral. When asked to describe feelings after referral to the hospital (*n* = 240 responses), 74.6% of the responding patients experienced negative emotions at that time (16.7% neutral, 8.8% positive) (Figure 4).

#### Recommendations to Patients

Overall, the advice and tips offered by patients to those undiagnosed or living with pulmonary fibrosis were: seeking help early when you experience symptoms, pushing for a speedier diagnosis, seeking as much information as possible from healthcare professionals at all stages, taking regular exercise, joining pulmonary rehabilitation classes to assist with breathlessness, joining patient support groups, remaining positive, pacing themselves, and making the most of their time. General tips for fellow patients regarding mental well-being contained phrases such as: stay calm, stay positive, no stress, don't despair, don't give up, focus on the present, and don't get agitated, frustrated or anxious.

#### Recommendations to Healthcare

Advices to healthcare workers included performing tests earlier, providing more information and lifestyle advice, gaining more knowledge about pulmonary fibrosis, improving communication between healthcare workers, structuring the diagnostic process better, and earlier start of pharmacological and palliative treatment. More recommendations are listed as quotes in Supplementary Material 4.

## Discussion

The purpose of this survey was to document the time taken to diagnosis and to identify potential causes of delays at different stages of the diagnostic pathway for pulmonary fibrosis patients in Europe. The second aim was to describe patients' experiences during this journey.

We found that the time to diagnosis varies widely. Only 30% of patients were diagnosed with pulmonary fibrosis within 3 months of symptom onset, while for over 40% of patients it took more than 1 year to be diagnosed. Other studies observed a median time from onset of first symptoms to diagnosis of 7 months (range 0–252) based on a patient survey (9) and 2.1 years (IQR 0.9–5.0) from a retrospective cohort study (5). In 2020, a group of ILD specialists reported a mean time from symptom onset to pulmonary fibrosis diagnosis of 2.3 years (Q1–Q3: 2–3) (19). The proportion of patients in our cohort who took more than a year to be diagnosed is smaller than that reported by other studies of pulmonary fibrosis patients (9, 11). Moreover, in a study of IPF patients, the median time to diagnosis was 13.6 months (range 5.9–39.5; max. 274.3) but 49% of the cohort received a diagnosis after more than 1 year (17). In another study, the median time for establishing a diagnosis was 1.5 years (range <1 week to 12 years) but this was calculated from the time of the first doctors' appointment rather than onset of symptoms (12). Compared to these historical studies, our results suggest fewer patients had such long delays from symptom onset to diagnosis.

Delays in diagnosis can occur at each stage of the patient journey and may be due to both patient- and healthcare-related causes. The longest delay we observed occurred in stage 1 (Figure 1B). More delay in this stage translated into a prolonged time to the final diagnosis. Our results show that only a quarter (26.7%) of all patients did not delay their initial appointment with their primary care physician. These findings are similar to results from a patient survey conducted in 2015 (9). A more recent survey amongst IPF patients reported a median delay of 0.1 years for this stage (5). From our survey, those who delayed their appointment reported they had not been concerned about their symptoms. This highlights the need to raise awareness of pulmonary fibrosis amongst the general public, so that individuals seek medical assistance earlier.

The time taken by people being treated in primary care (stage 2) varies. In our survey, almost 40% of patients were referred to a hospital specialist after their first primary care appointment, which is greater than that observed in a study conducted in the USA in 2015 (27.8%) (9). However, Hoyer et al. found that 80% of patients in Denmark (between 2016 and 2019) were referred after 1 or 2 visits to their general practitioner (5). These observations may reflect differences in healthcare systems or in awareness of pulmonary fibrosis between countries.

Of all respondents, 15.3% were referred after 4 or more appointments. Several factors may contribute to delays in primary care. Firstly, initial symptoms in the early stage of the disease can be non-specific and not yet known to be life threatening. In support of this, 42% of IPF patients had a normal lung function when initially assessed in primary care (18). Secondly, primary care physicians may suspect the symptoms to be due to more common respiratory diseases (such as asthma, pneumonia, bronchitis, allergies, and COPD [9]) and decide on a period of observation (20). Such misdiagnosis occurs in up to 41% of patients (5) and can prolong time to establish an ILD diagnosis (9, 10). Thirdly, primary care physicians may lack knowledge about pulmonary fibrosis. A study in Finland found almost half of referral letters lack key information related to possible ILD diagnosis (18). An e-learning for General Practitioners has recently been launched by the Royal College of General Practitioners in the United Kingdom and patient organisation APF to increase knowledge about symptoms and treatment of pulmonary fibrosis (21). In other countries, similar initiatives are evolving.

Stage 3 is the time between being referred and the patient's actual hospital appointment. Based on our data, 76.9% were assessed by a pulmonologist within 3 months, compared to 91% reported from a Finnish cohort (18). In this Finnish study only referral letters to tertiary care centres were evaluated, which may explain the higher percentage. However, in the United Kingdom and Ireland the time to secondary care respiratory clinic visit [47 days (25–84)] was significantly less than the time to an ILD specialist clinic visit [290 days (133–773)] (16). Given differences in the structure and complexities of healthcare systems, it is difficult to compare data from different countries. To our knowledge, there are no published data as to why delays in stage 3 occur. It may reflect waiting times or patients postponing a hospital clinic appointment.

Delays occurring from the first hospital appointment to final diagnosis (stage 4) can be partly explained by the number of diagnostic tests, access to them (22) and challenges in confirming a specific diagnosis accurately. Patients in our survey underwent on average 4.5 tests per person. The most common were spirometry, blood tests, and radiological chest imaging, similar to those reported by others (9, 14). The proportion of reported lung biopsies was surprisingly high in our cohort (41.9–67.9%), which may reflect variation in healthcare practises, as biopsy rates differ between countries [16.1–1.2% (2013–2019) in England (23), 34.1% in Germany (2012–2014) (24), 20.1% in Italy (2015–2017) (25)].

Several parameters may predict potential delays, as they are associated with an increased time to diagnosis. In our cohort patients with a final diagnosis of IPF experience shorter delays and undergo less invasive diagnostic testing than patients with other diagnoses. These differences may be due to IPF patients presenting with more severe symptoms initially, availability of the IPF international diagnostic guidelines, or availability of tests (22, 26). We can only speculate on this as we did not collect data on disease severity nor have powered for separate subgroup analyses. Another parameter that may influence time to diagnosis are the specific presenting symptoms. When patients present with dyspnoea, the median time to confirm an ILD diagnosis was 307 days, which increased for symptoms as cough and fatigue, to 563 and 639 days, respectively (15). Similarly, Pritchard et al. found an association between dyspnoea and a shorter time to hospital referral, which was not observed for lung crackles or chronic cough (8). Other factors that may contribute to a delayed diagnosis include presence of specific comorbidities, male sex, increased body mass index, older age, previous inhalation therapy use, preserved diffusing capacity and better St. George's Respiratory Questionnaire scores (5, 7, 16, 17). Lastly, abnormal chest imaging is one of the main reasons to initiate a hospital referral from primary care (8, 18) and naming ILD on the thoracic CT radiologic report doubled the likelihood of a referral to a pulmonologist within 6 months (8). Interestingly, performing lung function tests in primary care, which indicated the possibility of ILD did not significantly influence time to CT scan or hospital referral (8).

### Patients' Experiences

The pulmonary fibrosis journey to diagnosis generally involves extensive, repetitive, and sometimes invasive testing. Most patients in the survey reported that this causes a considerable burden, which can impact on emotional health, finances, and personal and professional life (9). Shortening the diagnostic journey and assessment at an ILD expert centre results in higher patient satisfaction (12). In addition, our survey highlighted the need to better inform patients during their diagnostic journey, to provide information on how to live with pulmonary fibrosis and advice on lifestyle changes at diagnosis. After diagnosis, providing information on perspectives, and options and discussions concerning symptom management should also be a priority as identified by our respondents. These observations are similar to those reported from surveys and in-depth patient interviews (27, 28). In one paper, authors highlighted that patients need time to come to terms with their diagnosis and that repeated provision of information was essential to fully understand the consequences and implications of their disease (11). However, a survey of ILD professionals in Europe showed that although two-thirds of specialist centres offered patient education only a few patients attended these existing programmes (10). Furthermore, only 6% of patients from our survey were informed about support groups, despite the value of peer support to patients and carers reported not only by our respondents but also from a previous patient survey (12). However, scientific evidence for the benefits of peer support is scarce (29). Regarding caregivers' needs, several patients in our survey highlighted the need to provide them with more information on the patient's experience and practical help on how best to support them (30). Finally, providing details of websites which offer reliable and accurate information is important as many websites contain incorrect or out-dated information (31).

### Limitations

In this study, we used a variety of survey methods, which resulted in a good understanding of patients' perceptions and experiences. Nevertheless, using patient reported data is also a weakness of this study. A general limitation of open-ended questions is the variety of responses, which could not be included in the quantitative analysis. Limitations also include patient recall, non-response, and misinformation bias. These factors could have influenced the lung biopsies reported in our cohort, as patients may not differentiate between procedures such as endobronchial biopsies, surgical biopsies, or only bronchoscopy. As the responses were anonymous, we could not confirm information from medical records.

Several factors prevent generalisation of these results to the overall population of patients with pulmonary fibrosis. We used a non-random sample of self-selecting pulmonary fibrosis patients invited via patient associations without a pre-defined number of invited patients, target, or countries. Most organisations have, until recently, focused on supporting and representing IPF patients, which likely accounts for the high number of IPF participants in this survey. Furthermore, patient characteristics, such as gender, age, comorbidities, and stage and/or severity of disease were not collected.

Although there are European guidelines for the diagnostic pathway of IPF and other ILDs, differences exist between countries (10). This may be related to the organisation of healthcare and options for primary care physicians to refer for CT scans or to ILD expertise centres. In our survey, we did not take these differences into account nor collect information on whether a CT chest scan was performed in primary care.

### Recommendations Clinical Practice

There is an urgent need to improve the diagnostic journey and recommendations on how to achieve this have been raised in several papers (10, 12, 13). Our findings on patient satisfaction and diagnostic delay endorse this and encourage further improvement. Rapid diagnosis is becoming increasingly important because several treatments are currently available to slow disease progression, improve quality of life, and may extend life expectancy (32–34). Although there are guidelines and other guidance documents on features, diagnosis, and management of ILD (26, 35–37) many patients have a diagnosis that is not confirmed by a multidisciplinary discussion and do not receive treatment (38). Additionally, geographical differences that may influence time to diagnosis and access to treatment still exists between countries (10).

In Table 2, we provide concrete strategies for each stage of the diagnostic journey to improve the standard clinical practise and patient satisfaction in order to promote a more rapid pathway for patients with pulmonary fibrosis throughout Europe. These strategies are based upon our survey outcomes, available literature, and expert authors' opinions. Awareness and education in general public, patients, and healthcare workers is a major topic in this field, as well as for other rare lung diseases (40).

**Table 2:** Strategies for improving the diagnostic pathway of pulmonary fibrosis patients.

	**Stage 1**	**Stage 2**	**Stage 3**	**Stage 4**	**After diagnosis**
Education and information	Increase awareness of PF amongst the general public.	Increase awareness of PF symptoms amongst primary care physicians and nurses.	Inform patients and policy makers on the need for urgency in hospital referral.	Inform patients about the reasons for diagnostic investigation and the differential diagnosis.	Inform patients about drug treatment, non-pharmaceutical treatment (rehabilitation, oxygen therapy, palliative care, lung transplant), prognosis and lifestyle
Improving standard care		Develop criteria for referral for chest CT scan or to a specialist when abnormalities on examination suggest PF.	Regular (virtual) MDDs between general hospital specialist and ILD experts.	Day case assessment with diagnostic investigations and clinical assessment.	Introduce psychological support, helplines and peer groups for patients as part of standard care.
		Better communication between primary care physician and ILD specialist.	Increase the number of ILD specialists in general hospitals.	Availability of DLCO measurement in all hospitals.	Discuss duration and frequency of follow-up visit.
Research areas	Identify the optimum way to provide information about PF to the general population.	Cost-effectiveness of performing chest CT scan in primary care or at community facilities.	Comparing waiting times and diagnostic pathway of PF to other uncommon diseases or disorders with poor prognosis [e.g., cancer (39)].		Assess caregivers' needs on counselling and support.

*Content is based on survey outcomes, available literature, and authors' opinions. CT, computed tomography; DLCO, diffusing capacity for carbon monoxide; ILD, interstitial lung disease; IPF, idiopathic pulmonary fibrosis; MDD, multidisciplinary discussion; PF, pulmonary fibrosis*.

## Conclusion

From the onset of symptoms to diagnosis of pulmonary fibrosis, the patient journey involves delays at each stage of the diagnostic pathway. Most of these delays are avoidable. Based upon our findings, there is a particular need to raise awareness of pulmonary fibrosis in the general population. Additionally, patients' experiences highlight the need for understandable information concerning the diagnostic tests performed, differential diagnosis, final diagnosis, and treatments as well as peer support groups. Improving several aspects of the diagnostic pathway for pulmonary fibrosis is therefore warranted to minimise delays and improve patient satisfaction throughout Europe.

## Data Availability Statement

The raw data supporting the conclusions of this article will be made available by the authors, without undue reservation.

## Ethics Statement

Ethical review and approval was not required for the study on human participants in accordance with the local legislation and institutional requirements. The patients/participants provided their written informed consent to participate in this study.

## Author Contributions

IvS organised the database and performed the statistical analysis. IvS and SJ wrote the first draught of the manuscript. IvS, SJ, DC, and HP wrote sections of the manuscript. All authors contributed to conception and design of the study and contributed to manuscript revision, read, and approved the submitted version.

## Conflict of Interest

IvS reports grants from Boehringer Ingelheim outside the submitted work. SJ reports grants from Action for Pulmonary Fibrosis during the conduct of the study. FB reports personal fees and other from Boehringer Ingelheim, Roche, Galapagos, Savara outside the submitted work. AS reports personal fees from Roche, Boehringer Ingelheim, Berlin Chemie Menarini, Teva-Actavis, Medopharma, S&D Pharma Logistics and Pfizer, and grants from GlaxoSmithKline and Berlin Chemie Menarini outside the submitted work. IvS reports personal fees from Galapagos, Boehringer Ingelheim, Roche Pharma, Novartis Pharma, Menarini and Astra Zeneca outside the submitted work. MW reports grants and other from Boehringer Ingelheim and Hoffman la Roche, and other from Respivant, Galapagos, Safara, Novartis and Bristol Meyer Squib outside the submitted work. All grants and fees were paid to MW's institution. HP reports consultancy fees and conference travel from Roche and Boehringer Ingelheim; an educational grant and speaker fees from Roche. HP is a trustee for Action for Pulmonary Fibrosis and a member of the EU-IPFF scientific advisory board. KL reports personal fees, travel grants and consultancy fees from Boehringer Ingelheim and Roche outside submitted work. The remaining authors declare that the research was conducted in the absence of any commercial or financial relationships that could be construed as a potential conflict of interest.

## Publisher's Note

All claims expressed in this article are solely those of the authors and do not necessarily represent those of their affiliated organizations, or those of the publisher, the editors and the reviewers. Any product that may be evaluated in this article, or claim that may be made by its manufacturer, is not guaranteed or endorsed by the publisher.
