# Supplementary material for: Patient Reported Experiences and Delays During the Diagnostic Pathway for Pulmonary Fibrosis: A Multinational European Survey
Source: Front Med (Lausanne). 2021 Aug 4;8:711194. doi: 10.3389/fmed.2021.711194 (PMC8371687; doi:10.3389/fmed.2021.711194)
Supplement: Supplementary file 1 [file Data_Sheet_1.docx]

Supplementary Material

# Survey: Uncovering insights into the pulmonary fibrosis patient journey

## Introductory questions

1. All of the information you have supplied in the survey is anonymous and your personal data will not be collected. Please confirm if you are happy to receive a report of the survey including key findings and top tips shared by other patients.
   1. I accept
   2. I don’t accept
2. Where are you from/which healthcare system did you go through?
   1. UK
   2. Ireland
   3. France
   4. Spain
   5. Italy
   6. Austria
   7. Belgium
   8. Bulgaria
   9. Poland
   10. The Netherlands
   11. Hungary
   12. Norway
   13. Germany
   14. Other
3. What is your diagnosis?
   1. Idiopathic pulmonary fibrosis
   2. Chronic hypersensitivity pneumonitis
   3. Autoimmune-related pulmonary fibrosis (e.g. scleroderma or rheumatoid related)
   4. Sarcoidosis
   5. Non classified
   6. Other

## At home

1. When thinking about your journey to a diagnosis of pulmonary fibrosis, which symptoms were you experiencing before making an initial doctor’s appointment? Tick as many as apply.
   1. Dry cough
   2. Shortness of breath
   3. Acid reflux
   4. Feeling more tired than usual
   5. Loss of appetite/weight loss
   6. Rounded/swollen fingertips
   7. Aching muscles/joints
   8. Other
2. Did you visit your pharmacist prior to visiting your doctor?
   1. Yes
   2. No
3. How long did you wait from the onset of your symptoms to making an appointment with your doctor?
   1. Less than 1 week
   2. Less than 1 month
   3. 1 to 3 months
   4. 3 to 6 months
   5. 6 months to 1 year
   6. 1 year to 2 years
   7. Over 2 years
   8. Don’t know/can’t remember
4. If you delayed going to see your doctor, why? Tick as many as apply.
   1. You thought your symptoms were related to your age
   2. You didn’t want to be a burden to your doctor as they are busy
   3. Your symptoms didn’t cause you concern
   4. You were worried what the doctor might say
   5. You did not delay
   6. Other
5. What prompted you to finally go? Tick as many as apply.
   1. You were worried about your cough
   2. You were worried about your shortness of breath
   3. You were worried about feeling more tired than usual
   4. Your loss of appetite/weight loss
   5. Your painful/aching muscles/joints
   6. A family member/friend suggested you go
   7. Your symptoms were impacting on your daily activities (at home or work; please give details below after clicking ‘OK’)
   8. You don’t know/can’t remember
   9. Other
6. Your symptoms were impacting on your daily activities (at home or work; please give details here).
7. What were your feelings at this stage of your journey?
8. Is there anything that could have helped you at this stage?

## General practitioner/primary care practice

1. What happened during your first appointment at your general practitioner/primary care practice, and what was the approach recommended by your doctor? Tick as many as apply.
   1. I was examined but no action was taken, and was told to come back if my symptoms continued or worsened
   2. The doctor performed some tests, but I stayed within their care
   3. I was diagnosed with and treated for another condition, e.g. asthma, chronic obstructive pulmonary disease, heart failure
   4. I was referred to a pulmonologist in a hospital
   5. I was referred to another specialist in a hospital (please provide further details after clicking ‘OK’)
   6. I don’t know/can’t remember
   7. Other
2. I was referred to another specialist in a hospital (please provide further details below).
3. How many times did you see your general practitioner before you were referred to a hospital?
   1. Once
   2. Two to three times
   3. Four to five times
   4. Over six times
   5. Don’t know/can’t remember
4. How long did it take until you saw a specialist at a hospital from when your doctor referred you?
   1. Less than 1 week
   2. Less than 1 month
   3. 1 to 3 months
   4. 3 to 6 months
   5. 6 months to 1 year
   6. 1 year to 2 years
   7. Over 2 years
   8. Don’t know/can’t remember
5. What were your feelings at this stage of your journey?
6. What worked well or what could have helped you?

## Hospital setting

1. What investigations/tests did you undergo at the hospital? Tick as many as apply.
   1. Lung function test (spirometry)
   2. Blood tests
   3. Chest x-ray
   4. Computed tomography (CT) scan/lung imaging
   5. Lung biopsy
   6. Lung wash (lavage)
   7. 6-minute walk test
   8. Don’t know/can’t remember
   9. Other
2. Were you told why these different tests were needed?
   1. Yes
   2. No
3. Do you feel you were given sufficient information?
   1. Yes
   2. No
4. What information would have been helpful?
5. In addition to your respiratory doctor, which other healthcare professionals did you see at the hospital? Tick as many as apply.
   1. Respiratory nurse
   2. Cardiologist
   3. Radiologist
   4. Don’t know/can’t remember
   5. Other
6. Which of the following correctly describes the outcome of the tests at the hospital?
   1. Diagnosed with pulmonary fibrosis at this hospital
   2. Referred to another hospital that specializes in pulmonary fibrosis
   3. Don’t know/can’t remember
7. What information did you receive at this stage in your journey?
8. What additional information do you wish you had been given?
9. How long did it take for you to be given a confirmed diagnosis of pulmonary fibrosis from your first appointment at a hospital?
   1. Less than 1 week
   2. Less than 1 month
   3. One to 3 months
   4. 3 to 6 months
   5. 6 months to 1 year
   6. 1 year to 2 years
   7. Over 2 years
   8. Don’t know/can’t remember
10. What information were you provided with at diagnosis, and after?
    1. Explanation by the doctor and/or specialist nurse during consultation
    2. Printed educational materials
    3. Educational materials to help explain my diagnosis to friends/family
    4. Support group recommendation
    5. Website recommendation
    6. Don’t know/can’t remember
    7. Other
11. What did you find especially helpful?
12. What could have been helpful for you and your carers?
13. When thinking about your entire journey, from first symptoms to your diagnosis, how long did it take?
    1. Less than 1 week
    2. Less than 1 month
    3. One to 3 months
    4. Three to 6 months
    5. 6 months to 1 year
    6. 1 year to 2 years
    7. 2 years to 5 years
    8. Over 5 years
    9. Don’t know/can’t remember

## To help other patients with pulmonary fibrosis

1. What piece of advice would you give to patients navigating the route to diagnosis in future?
2. What top tips could you provide on adjusting your lifestyle to live with pulmonary fibrosis?

Thank you for taking the time to complete this survey. Your input and insights are extremely valuable.
Watch out for a future email in which we will circulate key findings and share the top tips provided by everyone who has taken part.

# Literature search

*Embase.com*

('delayed diagnosis'/de OR (((delay* OR time-to) NEAR/3 diagnos*)):ab,ti) AND ('interstitial lung disease'/exp OR 'lung fibrosis'/exp OR 'lung sarcoidosis'/de OR ((interstitial* NEAR/3 (lung OR pulmonary*) NEAR/3 disease*) OR ((eosinophil* OR interstitial*) NEAR/3 pneumon*) OR (idiopathic* NEAR/3 (lung OR pulmonary*) NEAR/3 fibros*) OR (fibros* NEAR/3 alveolit*) OR (ANCA NEAR/3 vasculitide*) OR (Wegener* NEAR/3 granulomato*) OR ((lung OR pulmonary*) NEAR/3 sarcoidos*)):ab,ti) AND [english]/lim NOT ([animals]/lim NOT [humans]/lim)

*Medline ALL Ovid*

(Delayed Diagnosis / OR (((delay* OR time-to) ADJ3 diagnos*)).ab,ti.) AND (exp Lung Diseases, Interstitial/ OR Pulmonary Fibrosis/ OR Sarcoidosis, Pulmonary/ OR ((interstitial* ADJ3 (lung OR pulmonary*) ADJ3 disease*) OR ((eosinophil* OR interstitial*) ADJ3 pneumon*) OR (idiopathic* ADJ3 (lung OR pulmonary*) ADJ3 fibros*) OR (fibros* ADJ3 alveolit*) OR (ANCA ADJ3 vasculitide*) OR (Wegener* ADJ3 granulomato*) OR ((lung OR pulmonary*) ADJ3 sarcoidos*)).ab,ti.) AND english.la. NOT (exp animals/ NOT humans/)

*Web of science (Science Citation Index Expanded & Social Sciences Citation Index)*

TS=(((((delay* OR time-to) NEAR/2 diagnos*))) AND (((interstitial* NEAR/2 (lung OR pulmonary*) NEAR/2 disease*) OR ((eosinophil* OR interstitial*) NEAR/2 pneumon*) OR (idiopathic* NEAR/2 (lung OR pulmonary*) NEAR/2 fibros*) OR (fibros* NEAR/2 alveolit*) OR (ANCA NEAR/2 vasculitide*) OR (Wegener* NEAR/2 granulomato*) OR ((lung OR pulmonary*) NEAR/2 sarcoidos*)))) AND LA=(english)

*Cochrane CENTRAL register of trials*

((((delay* OR time next to) NEAR/3 diagnos*)):ab,ti) AND (((interstitial* NEAR/3 (lung OR pulmonary*) NEAR/3 disease*) OR ((eosinophil* OR interstitial*) NEAR/3 pneumon*) OR (idiopathic* NEAR/3 (lung OR pulmonary*) NEAR/3 fibros*) OR (fibros* NEAR/3 alveolit*) OR (ANCA NEAR/3 vasculitide*) OR (Wegener* NEAR/3 granulomato*) OR ((lung OR pulmonary*) NEAR/3 sarcoidos*)):ab,ti)

*Google Scholar*

"delayed diagnosis"|"time to diagnosis"|"diagnostic delay" "interstitial|idiopathic lung|pulmonary disease|fibrosis"|"eosinophilic|interstitial pneumonia"|"fibrosing alveolitis"|"lung|pulmonary sarcoidosis"

## Result

*Number of papers Number after deduplication*

*Embase.com* 764 753

*Medline ALL Ovid* 717 524

*Web of science** 176 34

*Cochrane CENTRAL register* 4 0

*Google Scholar*  200 128

***Total*  1861 1439**

*(Science Citation Index Expanded & Social Sciences Citation Index)

# Reported feelings

Feelings in period after onset of first symptoms (not displayed in figure)

**Coded described feeling Count**

Worried 20

Concerned 18

Fear 11

Uncertainty 7

Frustration 5

Discouraged 4

Distress 4

Surprised 3

Terrible 2

Disbelief 2

Panic 2

Anxiety 2

Unsure 2

Scared 2

Sadness 2

Helpless 2

Bad 1

Uneasy 1

Angry 1

Bewildered 1

Unwell 1

Frightened 1

Shocked 1

Annoyed 1

Discomfort 1

Guilty 1

Unsettled 1

Healthy 1

Dissatisfied 1

Heart problems 1

Despair 1

Anger 1

Disappointed 1

Incredulity 1

Troublesome 1

Innovative 1

Uncomfortable 1

Misunderstood 1

Unsatisfied 1

Not right 1

Apprehension 1

Confident 1

Weak 1

Perfect 1

Powerless 1

Confused 1

***Grand Total 118***

Feelings in period after referral to hospital (displayed in Figure 6)

**Coded described feeling Count**

Worried 47

Concerned 21

Anxiety 10

Fear 9

Uncertainty 9

Nervous 4

Good 4

Confused 4

Fine 4

Disappointed 4

Helpless 4

Annoyed 3

Bad 3

Afraid 3

Distressed 3

Frustrated 3

Not right 2

Inquisitiveness 2

Frightened 2

Terrible 2

Misunderstood 2

Alone 2

Scared 2

Disbelief 2

Curious 2

Uneasy 2

Collapsed world 2

Hopefull 2

Vulnerable 1

Discomfort 1

Bored 1

Great 1

Frustration 1

Healthy 1

Resigned 1

Doubts 1

Stressful 1

Helplessness 1

Trust 1

Hope 1

Unsure 1

Hopeful 1

Doubt 1

Dread 1

Sadness 1

Desperate 1

Shocked 1

Exhausted 1

Surprised 1

Expectation 1

Tired 1

Mixed feelings 1

Devastated 1

Angry 1

Unsettled 1

No understanding 1

Upset 1

Devestated 1

Well 1

Overwhelmed 1

Relieved 1

Incredulous 1

***Grand Total 193***

# Recommendations and experiences

Supplementary Table 1: Examples of recommendations from patients to healthcare providers (quotes from survey responses)

| **Recommendations for diagnostic pathway** | | | **Positive experiences** |
| --- | --- | --- | --- |
| **Before first GP visit** | **After referral to hospital** | **At time of diagnosis** | **During diagnostic pathway** |
| Get an earlier appointment | Earlier referral to specialist center | More extensive information about what to expect | Taking rest |
| Earlier referral to hospital | Earlier appointment and testing | Offering psychological help | Receiving treatment with antibiotics, steroids, oxygen or antifibrotics |
| Getting the correct diagnosis | Earlier diagnosis | Making aware of patient associations and support groups | Support or concern from partner and family |
| Earlier start of treatment | Earlier start of (palliative) treatment | Advice regarding lifestyle | Adequate doctor: quick referral, information and discussion |
| More extensive examination | Getting more information from doctor | Advice on how to manage symptoms | Staying calm and positive |
| Getting more information and answers from doctor | More explanation by and discussion with doctor |  | Change specialist |
| Doctor with more knowledge about PF | Doctor with more knowledge about PF |  | Exercise |
| More knowledge about PF in general population | IPF training for general practitioners |  | Diagnostic testing during hospitalization |
| Better coordination between doctors | More knowledge about PF in general population |  | Information from doctor |
| Lifestyle advice from doctors (e.g. Lose weight, quit smoking) | Psychological help |  | Adequate referral to specialist center |
|  | More structured approach to diagnosis |  | Fast testing |
|  | Semi-annual lung function test |  | Participation in a clinical trial |
|  | Concerned doctor |  |  |

Supplementary Table 2: Examples of recommendations from patients to future patients (quotes from survey responses)

| **Advice to future patients** | |
| --- | --- |
| **During diagnostic pathway** | **Lifestyle after PF diagnosis** |
| Make an appointment with a GP sooner | Keep moving |
| Take enough rest | Have a healthy diet |
| Look for psychological support (professional psychologist or peer groups) | Look for (psychologic) support |
| Look for psychological support for family | Stay positive |
| Make early/immediate appointment when (persisting) symptoms of cough or shortness of breath | Continue life as normal as possible |
| Feel comfortable with your doctor, don’t hesitate asking for a second opinion | Adjust your pace when listening to your body |
| Consult other doctor if you don’t feel comfortable or taken seriously | Accept your limitations, but don’t lock yourself at home |
| Ask questions and explanation about the tests, disease and therapy | Continue social activities |
| Start treatment (drugs, oxygen or transplant) as soon as possible | Find a balance with enough rest, but keep doing physical exercise as much as possible |
| Ask about ongoing clinical trials and new medication. | Do breathing exercises |
| Look up information yourself only in reliable sources | Do not overexert |
| Insist on further testing and/or referral | Find new hobbies |
| Go to specialised pulmonologist and hospital for adequate diagnosis | Quit smoking |
| Look for support groups and patient associations | Find support with fellow patients |
| Keep exercising | Look for psychological help |
| Join physiotherapy and/or rehabilitation courses |  |
| Live healthy, eat good food |  |
| Listen to the doctor’s advice |  |
| Look for professional psychological support |  |
| Look for good people around you |  |
| Distraction |  |
